# P-84. Periprosthetic infections: Treatment failure and mortality

**DOI:** 10.1093/ofid/ofaf695.313

**Published:** 2026-01-11

**Authors:** Gustavo A Castro Torres, Elena Temporiti, Pablo Bonvehí, Fabián Herrera, Diego Torres, Roger Torga, Eduardo Abalo, Roberto Valentini, Nicolás Lasserre, Agustina Fiori, Julia Ramponi, Maximiliano Gabriel Castro, Marcia Querci

**Affiliations:** CEMIC: Centro de Educacion Medica e Investigaciones Clinicas Norberto Quirno, Ciudad autónoma de Buenos Aireis, Ciudad Autonoma de Buenos Aires, Argentina; Hospital Universitario CEMIC, Buenos Aires, Ciudad Autonoma de Buenos Aires, Argentina; CEMIC: Centro de Educacion Medica e Investigaciones Clinicas Norberto Quirno, Ciudad autónoma de Buenos Aireis, Ciudad Autonoma de Buenos Aires, Argentina; Centro de Educación Médica e Investigaciones Clínicas, CEMIC, BUENOS AIRES, Ciudad Autonoma de Buenos Aires, Argentina; Centro de Educación Médica e Investigaciones Clínicas, CEMIC, BUENOS AIRES, Ciudad Autonoma de Buenos Aires, Argentina; CEMIC: Centro de Educacion Medica e Investigaciones Clinicas Norberto Quirno, Ciudad autónoma de Buenos Aireis, Ciudad Autonoma de Buenos Aires, Argentina; CEMIC: Centro de Educacion Medica e Investigaciones Clinicas Norberto Quirno, Ciudad autónoma de Buenos Aireis, Ciudad Autonoma de Buenos Aires, Argentina; CEMIC: Centro de Educacion Medica e Investigaciones Clinicas Norberto Quirno, Ciudad autónoma de Buenos Aireis, Ciudad Autonoma de Buenos Aires, Argentina; CEMIC: Centro de Educacion Medica e Investigaciones Clinicas Norberto Quirno, Ciudad autónoma de Buenos Aireis, Ciudad Autonoma de Buenos Aires, Argentina; CEMIC: Centro de Educacion Medica e Investigaciones Clinicas Norberto Quirno, Ciudad autónoma de Buenos Aireis, Ciudad Autonoma de Buenos Aires, Argentina; CEMIC: Centro de Educacion Medica e Investigaciones Clinicas Norberto Quirno, Ciudad autónoma de Buenos Aireis, Ciudad Autonoma de Buenos Aires, Argentina; CEMIC: Centro de Educacion Medica e Investigaciones Clinicas Norberto Quirno, Ciudad autónoma de Buenos Aireis, Ciudad Autonoma de Buenos Aires, Argentina; Hospital Universitario CEMIC, Buenos Aires, Ciudad Autonoma de Buenos Aires, Argentina

## Abstract

**Background:**

Periprosthetic infections (PI) lead to increased morbidity, mortality, and healthcare costs. There are few data available on the various factors associated with treatment failure and mortality. The objective was to evaluate these factors in a specific patient cohort.

**Methods:**

Retrospective observational analytical study of patients diagnosed with PI of the hip and knee at a university hospital between 2014 and 2024.

Demographic variables, prosthesis type and indication, clinical data, isolated microorganisms, and treatment were assessed. Factors associated with treatment failure were defined as use of suppressive therapy (UST), second-look surgery (SLS), and mortality within one year of follow-up.

Measures of association were analyzed, including the Chi-square test (dichotomous variables) and Student's t-test (quantitative variables). A logistic regression model was used to determine mortality-associated factors.

**Results:**

Of 67 patients included in the study, 77% had hip PI and 23% knee PI. 34% were men, with a median age of 74 years (IQR 66–81). Ninety-five percent of the patients underwent surgery, 69% DAIR (debridement, antibiotics, and implant retention), and 21% two-stage implant replacement. Comorbidities included 13% diabetes, 22.3% immunosuppression, and 67% cardiovascular disease. Sixty-four percent had early PI (< 3 months) and 60% monomicrobial PI, with *Staphylococcus aureus* being the most frequently isolated microorganism (35%).

No association was found with the need for suppressive therapy. SLS was associated with loosening of a previous prosthesis (p=0.04), recurrent prosthetic dislocation (p=0.04), early infection (p=0.029), and DAIR (p=0.004). One-year mortality was 18%. Multivariate analysis revealed that it was associated with fracture as an indication for prosthesis replacement (OR 7.21, p=0.002) and multidrug-resistant gram-negative bacilli (MDR-GNB) infection (OR 25.5, p=0.004).

**Conclusion:**

In our cohort of PI patients, treatment failure was associated with recurrent prosthetic loosening and dislocation, early infection, and DAIR, while mortality was associated with fracture as an indication for joint replacement and MDR-GNB infection.

**Disclosures:**

All Authors: No reported disclosures

